# Emotional text messages affect the early processing of emoticons depending on their emotional congruence: evidence from the N170 and EPN event related potentials

**DOI:** 10.1007/s10339-024-01223-y

**Published:** 2024-08-24

**Authors:** Nerea Aldunate, Vladimir López, Felipe Rojas-Thomas, Mario Villena-González, Ismael Palacios, Claudio Artigas, Eugenio Rodríguez, Conrado A. Bosman

**Affiliations:** 1 Laboratorio de Comportamiento Animal y Humano, Centro de Investigación en Complejidad Social, Facultad de Gobierno, Universidad del Desarrollo, Santiago, Chile; 2 Escuela de Psicología, Pontifica Universidad Católica de Chile, Santiago, Chile; 3 Center for Social and Cognitive Neuroscience (CSCN), School of Psychology, Universidad Adolfo Ibáñez, Santiago, Chile; 4 Centro de Estudios en Neurociencia Humana y Neuropsicología. Facultad de Psicología, Universidad Diego Portales, Santiago, Chile; 5 Departamento de Biología, Universidad Autónoma de Chile, Santiago, Chile; 6 Cognitive and Systems Neuroscience Group, Center for Neuroscience, Swammerdam Institute, University of Amsterdam, Amsterdam, The Netherlands; 7 Research Priority Program Brain and Cognition, University of Amsterdam, Amsterdam, The Netherlands

**Keywords:** N170, EPN, Early emotional processing, Emotional salience, Typographic emoticons, Context

## Abstract

Emoticons have been considered pragmatic cues that enhance emotional expressivity during computer-mediated communication. Yet, it is unclear how emoticons are processed in ambiguous text-based communication due to incongruences between the emoticon's emotional valence and its context. In this study, we investigated the electrophysiological correlates of contextual influence on the early emotional processing of emoticons, during an emotional congruence judgment task. Participants were instructed to judge the congruence between a text message expressing an emotional situation (positive or negative), and a subsequent emoticon expressing positive or negative emotions. We analyzed early event-related potentials elicited by emoticons related to face processing (N170) and emotional salience in visual perception processing (Early Posterior Negativity, EPN). Our results show that accuracy and Reaction Times depend on the interaction between the emotional valence of the context and the emoticon. Negative emoticons elicited a larger N170, suggesting that the emotional information of the emoticon is integrated at the early stages of the perceptual process. During emoticon processing, a valence effect was observed with enhanced EPN amplitudes in occipital areas for emoticons representing negative valences. Moreover, we observed a congruence effect in parieto-temporal sites within the same time-window, with larger amplitudes for the congruent condition. We conclude that, similar to face processing, emoticons are processed differently according to their emotional content and the context in which they are embedded. A congruent context might enhance the emotional salience of the emoticon (and therefore, its emotional expression) during the early stages of their processing.

**Supplementary Information:**

The online version contains supplementary material available at 10.1007/s10339-024-01223-y.

## Introduction

In the context of text-based communication through digital media, emoticons have been installed as a socio-affective pragmatic tool to express emotions (Aldunate and González-Ibáñez 2017; Derks et al. 2008). This has led to an increasing body of research exploring the influence of emoticons on communication. The evidence suggests that the insertion of emoticons into text messages not only brings more fun and intimacy to the communication (Hsieh and Tseng 2017; Huang et al. 2008; Thompson et al. 2016), but also enriches the emotional understanding of the communicants during text-based interactions (Filik et al. 2016; Walther and D’Addario 2001), helping them to discriminate emotions, attitudes, and intentions more accurately (Lo 2008; Rodrigues et al. 2017). Some studies have suggested that emoticons provide information that reduces language ambiguity during text-based communication (Kaye et al. 2017). For example, indirect messages with incongruent emoticons can be intended to express sarcasm (Holtgraves and Robinson 2020; Thompson and Filik 2016).

However, some studies have shown that the use of incongruent emoticons may complicate the interpretation of the sender’s mood (Aldunate et al. 2018a, b; Boutet et al. 2021). For example, in ambiguous communication such as sarcastic language, Howman and Filik (2020) found that wink emoticons preceded by positive messages were probably interpreted as sarcasm in young adults. Also, these emoticons were associated with increased text reading times compared to similar texts without emoticons, suggesting an augmented processing time of the message during reading. This evidence suggests that the preceding emotional context modulates the emoticons processing in a top-down manner. Thus, a relevant question to posit is whereby emotional contexts influence the emoticons processing; and particularly, how top-down processing can influence early emotional processing of the emoticons during text-based communication.

Emoticons share some electrophysiological correlates with faces. For instance, both faces and emoticons evoke a large N170 component, reflecting the earliest high-level visual processing of faces (Bentin et al. 1996; Rossion 2014). The N170 component is a negative-going deflection over occipital-temporal regions, with a maximum peak at 170 ms after face presentation (Eimer 2011). The N170 lateralization varies according to the visual stimulus category (Rossion and Jacques 2008; Rossion et al. 2003), with a right lateralization for faces (Campanella et al. 2000), a bilateral lateralization for objects (Rossion et al. 2000), and a left lateralization for linguistic stimuli (Maurer et al. 2008a, b). This difference suggests that early visual stimulus processing depends on their specific category, with different sources for this component (Itier and Taylor 2004; Maurer et al. 2008a, b).

Initially, the right N170 lateralization was considered an ERP related to the processing of the human faces structural features (Eimer et al. 2003; Herrmann et al. 2002). Nevertheless, recent studies have shown that the N170 amplitude can be influenced by other factors, such as the emotional expression of faces (Hinojosa et al. 2015). For example, it has been observed that faces expressing negative emotions evoke greater amplitudes for N170 compared to those conveying positive emotions (Batty and Taylor 2003). Thus, this modulation suggests that the N170 component does not only reflect the processing of invariant features of faces, but also the integration of the emotional expression in early perceptual processing of faces (Blau et al. 2007; Ibáñez et al. 2012; Kohler et al. 2000, 2003).

Other studies have shown that the N170 amplitude for human faces can be modulated by the context where faces are presented (Zhang et al. 2017). This effect has been observed in congruency paradigms where faces are presented in different scenarios or pictures (Wieser and Brosch 2012). Specifically, when participants are instructed to identify the emotion of faces, emotional expressions elicit larger N170 amplitudes when they are congruent with the emotional content of the scene (Diéguez-Risco et al. 2015; Hietanen and Astikainen 2013; Righart and de Gelder 2008a), suggesting that the situated emotional context facilitates the detection of the emotional expression of faces at the early stages of processing. Yet, the evidence on the influence of the linguistic context in N170 is inconclusive. For instance, Diéguez-Risco et al. (2015) observed that in a judgment congruence task, faces evoked larger amplitudes of the N170 component when they were emotionally incongruent with the linguistic context, as compared with those with emotionally congruent context. Conversely, and in a similar task, Dozolme et al. (2015) did not find congruence effects in the modulation of the N170. Similarly, Krombholz et al. (2007) did not observe congruence effects in the N170 modulation when participants saw happy and angry vertical schematic faces after the words "happiness" or "angry", while they had to count congruent trials, but they found enhanced amplitudes for this component when were angry faces.

Isolated typographic emoticons (for example, ‘:-)’) also evoke a right-lateralized N170 component similar to those observed during face processing (Churches et al. 2014). This modulation suggests that emoticons are recognized like faces, even if they are schematic compositions with a horizontal disposition (right to left). Furthermore, this modulation suggests that both emoticons and human faces share similar brain processing mechanisms. Cao and Zhao (2018) also observed this right-lateralization for four typographic emoticons, regardless of their emotional content and their congruency, expressing anger (‘ > :- < ’), surprise (‘:-o’), happiness (‘:-)’), and sadness (‘:-(’), all preceded by congruent or incongruent emotional faces.

Importantly, emoticons are usually preceded by linguistic contexts that sometimes convey emotional information. In the case of faces, contextual information is considered an important factor that influences their perceptual processing at different levels (Vinck and Bosman 2016; Aldunate et al. 2020; Ngo and Isaacowitz 2015; Righart and de Gelder 2008a). Recent studies have shown early effects of the emotional context during the processing of faces, reflected in the modulation of the Early Posterior Negativity (EPN) (Frühholz et al. 2009; Wieser et al. 2014).

The EPN component is a negative deflection over lateral occipital sites that tipically occurs between 150 and 300 ms after the presentation of emotional stimuli. This localization can extend to parieto-temporal regions depending on the nature and context of the visual stimulus (Schupp et al. 2003, 2006a, b). Previous studies have shown that both positively and negatively valenced stimuli elicit greater EPN amplitude compared to neutral stimuli (Kissler et al. 2009; Schupp et al. 2003), suggesting deeper early visual processing for these stimuli compared to neutral ones. Additionally, EPN amplitude is larger for more aroused emotional stimuli (Citron et al. 2013; Farkas et al. 2020). This increase in EPN amplitude for emotional stimuli has been interpreted as an indication of emotional facilitation in early perceptual processing, by more effective attentional resources allocation (Junghöfer et al. 2001, 2006; Schupp et al. 2004).

Interestingly, recent studies have found that faces preceded by congruent contexts in congruence judgement tasks elicit more negative deflections within the same EPN time-window at parieto-temporal regions (Diéguez-Risco et al. 2015; Dozolme et al. 2015). Thus, a larger EPN in congruent emotional stimuli could be reflecting a more facilitated allocation of attentional resources for the emotional stimuli processing, by the emotional information of the preceding context. All these suggest that text messages could serve as contextual information that might affect early processing of emoticons, influencing the amount of attentional resources involved in their emotional perceptual processing. Yet, the analysis of the early electro-cortical correlates of the influence that emotional context exerts during the emotional perception of emoticons remains unexplored.

In the present study, we analyze early ERPs during emoticons processing, presented after text messages referring to emotional situations in a congruency judgement task. We aimed to determine whether the affective information contained in the text-message, as context, influences the early processing of the emoticons. Specifically, the research questions that guide this study focus on how the emotional valence of emoticons and the preceding linguistic context influences the processing of emoticons as a face, analyzing the modulation of the N170 component (Bentin et al. 1996; Eimer 2011; Hadjikhani et al. 2009). On the other hand, this study aims to answer the question of how the emotional valence of the preceding linguistic context influences the emotional salience of the emoticon at the level of its early perceptual processing by facilitating attentional resources allocation, by analyzing the modulation of the EPN (Schupp et al. 2003, 2006a, b). In order to analyze how both the N170 and the EPN are modulated by the interaction between the emotional valence of the emoticon and the context, we implemented a congruency judgment task. We analyze the early ERPs (N170 and EPN) evoked by the presentation of positive and negative emoticons. All emoticons are preceded by text messages (linguistic contexts) that can also be of positive or negative valence, creating four experimental conditions: positive congruent (positive message–positive emoticon), negative congruent (negative message–negative emoticon), positive incongruent (negative message–positive emoticon) and negative incongruent (positive message–negative emoticon).

We hypothesize that, if the emotional valence of emoticons is integrated as when processing in the processing of human faces, their emotional valence should modulate the amplitude of the N170, reflecting early emotional integration. Furthermore, we hypothesize that if emotional content provided by prior linguistic context is integrated during the early perception of emoticons as a face, we might expect modulations in the N170 component due to a contextual effect. In relation to the EPN, we propose to explore how emotional valence and congruence/incongruence between the emoticon and the linguistic context modulate this component, considering its spatial and temporal dynamics. We expect that, if the emotional information from the preceding context influences the emotional salience of the emoticon, reflecting a greater allocation of attentional resources in its early perceptual processing, we will observe a greater amplitude of this component in congruent conditions. We recognize that emoticons, by themselves, carry emotional information and that the N170 is modulated by its emotional valence. However, our study focuses on how this modulation is influenced when emoticons are presented in different linguistic contexts. The novelty of our study lies in investigating whether and how the linguistic context affects the early perception of emoticons, not only through modulations of the processes reflected in the amplitude of the N170 component, but also through the modulations of the processes reflected in the EPN amplitude, something that has not been explored in depth in previous studies. This contributes to a greater understanding of the interaction between context and early emotional perception.

## Materials and methods

### Participants

Twenty undergraduate students voluntarily participated in the experiment (7 women, Mean age = 26 years + 1.8 SD). All of them were right-handed and Chilean native Spanish speakers. All participants had a normal or corrected-to-normal vision and none of them reported any history of neurological disease or psychiatric disorders. All participants signed the informed consent form, previously authorized by the Institutional Ethical Committee of BLINDED INSTITUTION.

### Materials

We created a set of 102 sentences written in Spanish and in the first person. All the sentences simulated text messages that indicate current personal situations that could be positive (e.g. “*They gave me a car*”) or negative (e.g. “*I crashed my car*”). First, to control the emotional valence and the emotional intensity of the sentences, we performed a pre-test. Sixty-eight participants (49 women, Mean age: 22.9 years ± 0.7 SD) were instructed to rate each sentence according to the perceived level of positivity or negativity, through a Likert scale (1–5), where 1 was ‘very negative’ and 5, ‘very positive’. Sixty sentences were selected based on their emotional valence ratings (see Supplementary Material). From this distribution, we took 30 sentences ranked as negative (Mean = 1.5; ± 0.2 SD) and 30 sentences ranked as positive (Mean = 4.6; ± 0.3 SD) (t(29) = 61.707; *p* < 0.001). Both lists contained the same level of emotional intensity (Mean_positive_ = 4.6; ± 0.3 SD; Mean_negative_ = 4.5; ± 0.3 SD; t(58) = 1.062; *p* = 0.290).

**Fig. 1:**
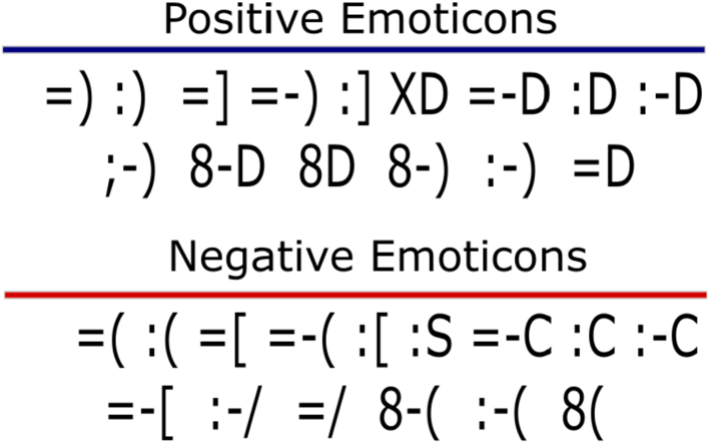
Set of emoticons used in this study. Fifteen typographic emoticons with positive valence (upper set) and fifteen typographic emoticons with negative valence (lower set)

Thirty typographical emoticons were presented in a horizontal left to right presentation using Calibri font (15 Positive Emoticons, 15 Negative Emoticons, see Fig. 1). To control the valence and emotional intensity of the emoticons, 75 participants (Mean age = 20.46; ± 0.4 SD; 47 women) were instructed to rate each emoticon according the perceived level of positivity or negativity, through a Likert scale (1–5), where again 1 was ‘very negative’ and 5, ‘very positive’. The ratings of valence for positive emoticons (Mean = 4.6; ± 0.2 SD) where significantly different compared to the negative emoticons (Mean = 1.8; ± 0.3 SD). Both sets of emoticons had the same level of emotional intensity (Mean_positive_ = 4.6; ± 0.2 SD; Mean_negative_ = 4.3; ± 0.4 SD; t(28) = 1.193; *p* = 0.24).

### Procedure

Participants sat 57 cm away from a computer screen with a gray background. The task was designed using Psychopy software (Peirce 2007). Every recording session involved 120 trials (60 congruent vs 60 incongruent), separated into two blocks of sixty trials each. After the first block, participants had voluntary resting time.

**Fig. 2:**
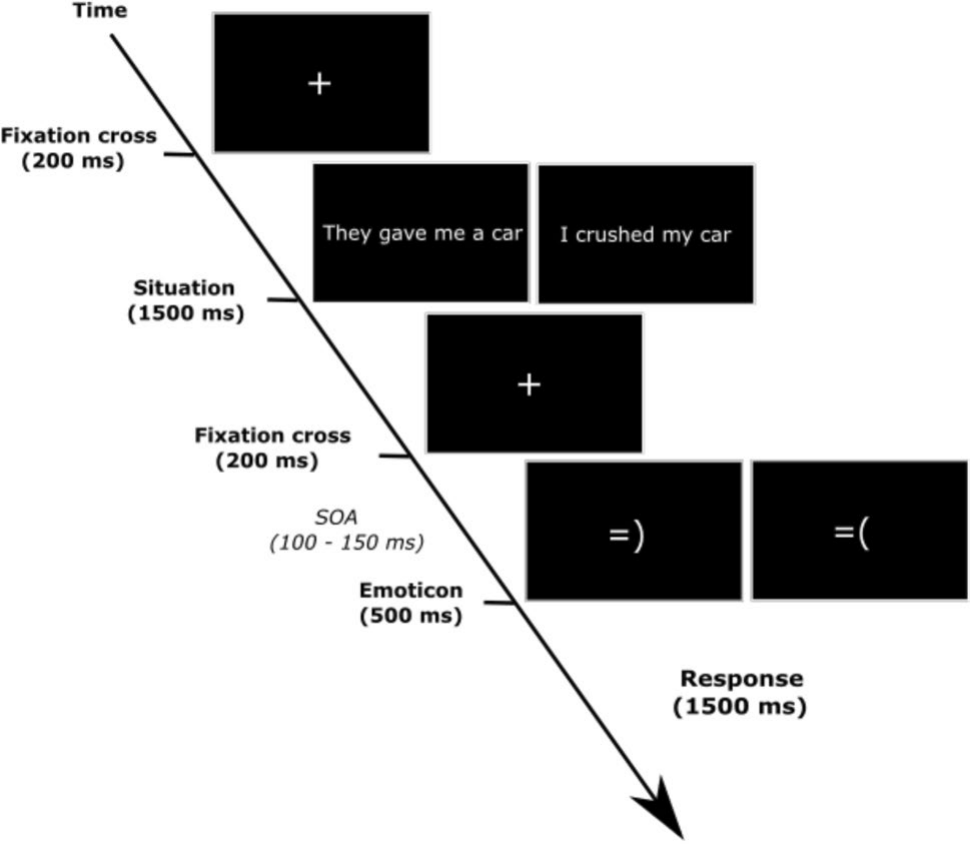
Experimental trial. Participants were instructed to judge the congruence between sentences presenting emotional situations (positive/negative) and subsequently presented emoticons (positive/negative)

During the task, the participants were asked to fix their eyes on a fixation cross in the middle of the screen and they were also asked to reduce their blinking as much as possible. Each trial began with a fixation cross, displayed for 200 ms on the screen. After fixation, a text sentence describing the emotional situation expressed in first-person was presented on the screen for 1,500 ms. After the presentation of the sentence, another fixation cross was displayed for 200 ms, followed by an inter-stimulus interval of 100–150 ms. Subsequently, an emoticon was presented for 500 ms. Once the emoticon disappeared, the screen was set to black (Fig. 2). Participants were instructed to press a keyboard key to judge the congruence of the emoticon according to the preceding text sentence within a time limit of 1500 ms. During the first experimental block, participants had to press the button “a” (with the left hand) of the keyboard if the emoticon was emotionally congruent with the sentence. In those conditions when emoticons were incongruent with the sentence, participants were prompted to press the button “l” (with the right hand) of the keyboard. To control for lateralized motor response effects, the response keys, and the hand to respond about congruence, were exchanged in the middle of the experiment (during the second experimental block).

All the sentences were presented twice, one time paired with a congruent emoticon and the other time paired with an incongruent emoticon. The stimuli were pseudorandomized to control that the same condition was presented only up to three times in a sequence.

### EEG recording and data pre-processing

EEG data was obtained with 64 electrodes (Biosemi® ActiveTwo) arranged according to the international 10/20 extended system. The data was processed and analyzed using the EEGLAB toolbox for MATLAB (Delorme and Makeig 2004). After the recording session, the data was resampled at 1000 Hz, demeaned and detrended. The EEG signals were subsequently filtered between 0.1 and 30 Hz and recalculated to the average reference. Two additional derivations were used (vertical electrooculogram; VEOG and horizontal electrooculogram; HEOG) to control for vertical and horizontal eye movements. All trials with amplitude fluctuations greater than 100 microvolts (mV), or values beyond 5 SD from the mean of the EEG signal, were considered contaminated by eye movements, blinks, and other artifacts, and were subsequently removed from further analysis. Participants had 1.5% rejected trials on average, with no difference among the four conditions (F(1,19) = 1.00; *p* = 0.330). Specifically, the positive congruent condition had 1.8 rejected trials (6.1%), the negative congruent condition had 1.6 rejected trials (5.5%), the positive incongruent condition had 2.0 rejected trials (6.6%), and the negative incongruent condition had 2.4 rejected trials (8.1%).

The EEG signal was segmented into epochs for each condition, from 200 ms preceding each stimulus onset up to 400 ms post-stimulus presentation. Epochs were sorted into four conditions (positive congruent, negative congruent, positive incongruent, and negative incongruent), and subsequently averaged across subjects and conditions. For each ERP component, we calculated the local peak and the mean amplitude according to the procedure described by Luck (2005).

The N170 peak was identified as the maximum negativity recorded voltage occurring between the 150 and 250 ms time window following stimulus onset. The N170 amplitude was defined as the 10 ms amplitude average around the peak for each participant and condition. The electrodes PO7 and PO8 were chosen for subsequent analyses, as they have been selected for the evaluation of the N170 amplitude in the literature (Rossion and Jacques 2008). The EPN amplitude was calculated as the mean amplitude between a 255–285 ms time-window, and from the occipital sites (POz, Oz, O1, and O2), as it has been reported previously (Schupp et al. 2006a, b; Yoon et al. 2016). Following the studies of Diéguez-Risco et al. (2015) and Dozolme et al. (2015), we analyzed the effects on the same temporal window at parieto-temporal sites (TP7, P7, CP5, P5, TP8, P8, CP6, and P6).

### Statistical analysis

The accuracy rates and RTs were considered for behavioral data analysis. The accuracy rate was calculated for each condition as the number of correct responses divided by the number of hits. RTs per trial (in milliseconds) were automatically calculated by the software Psychopy (Peirce 2007) during the task presentation. Subsequently, the accuracy rate and the RTs among conditions were compared by carrying out a repeated-measures ANOVA using congruence (congruent vs incongruent) and valence (positive vs negative) as factors.

The amplitudes of the N170 and EPN components at their specific time-window were compared between conditions performing a repeated-measures ANOVA using hemisphere (right vs left), congruence (congruent vs incongruent), and valence (positive vs negative) as factors. In all cases, the Mauchly’s Sphericity Test and the Greenhouse–Geisser corrections were applied when necessary. All the statistical analyses were assessed using the software STATISTICA 7.0 (StatSoft, Inc).

To further evaluate the effects of the different components across space and time, we performed a factorial univariate test using the Factorial Mass Univariate ERP Toolbox (FMUT) available from: https://github.com/ericcfields/FMUT/releases (Groppe et al. 2011a, 2011b). This analysis compares each electrode and latency at a region of interest, allowing temporally and topologically focused exploration of ERP dynamics with a priori specification. Following previous studies, we analyzed each electrode and time point followed by a permutation-based correction for multiple comparisons (Groppe et al. 2011a, 2011b). We used the following parameters: 10.000 permutations for a specific time window (103–298 ms), indicating an alpha cluster level of 0.05, and average of four neighbor channels for clustering.

## Results

### Behavioral results

Descriptive statistics for accuracy rate and RTs are provided in Table 1. The accuracy rate analysis show a main effect for the valence of the emoticons (*F*(1,19) = 11.04; *p* = 0.004, *η*^*2*^_partial_ = 0.367). Positive emoticons showed a better accuracy than negative emoticons. No significant differences were observed for congruence factor (F(1,19) = 3.72; *p* = 0.069, *η*^*2*^_partial_ = 0.163). Further, we observed an interaction between valence and congruence (*F*(1,19) = 6.90; *p* = 0.017, *η*^*2*^_partial_ = 0.266). Specifically, the accuracy was higher during positive congruence compared with negative congruence (*F*(1,19) = 10.62; *p* = 0.004, *η*^*2*^_partial_ = 0.358), while the incongruent condition showed no differences in accuracy comparing valence (*F*(1,19) = 1.866; *p* = 0.188). In addition, participants had a better accuracy when negative emoticons appeared after a positive context (incongruent condition) than they appeared after a negative context (congruent condition) (*F*(1,19) = 11.776; *p* = 0.003, *η*^*2*^_partial_ = 0.382). No differences were found for positive emoticons (*F*(1,19) = 0.954; *p* = 0.34).

**Table 1:** Behavioral descriptive statistics

		Congruent			Incongruent		
		Negative	Positive	Total	Negative	Positive	Total
Accuracy	%	80.71	93.22	86.97	88.5	90.77	89.64
	SD	13.8	7.98	12.8	10.29	9.38	9.79
	SEM	3.08	1.78	2.02	2.3	2.09	1.55
RT	Mean	0.607	0.438	0.516	0.552	0.557	0.554
	SD	0.10	0.10	0.09	0.11	0.09	0.09
	SEM	0.02	0.02	0.02	0.02	0.02	0.02

Accuracy rates (percentage of correct responses) and RTs (RT; in seconds) for congruent emoticons (negative vs positive valence; and total of congruent) and incongruent emoticons (negative vs positive valence; and total of incongruent). % = percentage, SD = standard deviation, SEM = standard error of the mean

A main effect of congruence was observed for RTs. We observed significantly faster RTs during congruent trials (*F*(1,19) = 5.56; *p* = 0.029, *η*^*2*^_partial_ = 0.226). A main effect was also found for valence, whereas positive showed faster RTs, rather than negative emoticons (*F*(1,19) = 52.88; *p* < 0.0001, *η*^*2*^_partial_ = 0.736). Furthermore, an interaction effect was observed between congruence and valence (*F*(1,19) = 64.62; *p* < 0.0001, *η*^*2*^_partial_ = 0.773). Specifically, in the congruent condition, RTs were faster when positive emoticons were presented (*F*(1,19) = 119.70; *p* < 0.0001, *η*^*2*^_partial_ = 0.863). We did not observe significant differences between positive and negative emoticons in the incongruent condition (*F*(1,19) = 0.137; *p* = 0.715). Incongruent conditions showed faster RTs than congruent ones when the emoticon valence was negative (*F*(1,19) = 11.43; *p* = 0.003, *η*^*2*^_partial_ = 0.376). Conversely, congruent condition showed faster RT than incongruent condition when the valence of the emoticons was positive (*F*(1,19) = 41.73; *p* < 0.0001, *η*^*2*^_partial_ = 0.687).

### ERP results

#### N170

**Fig. 3:**
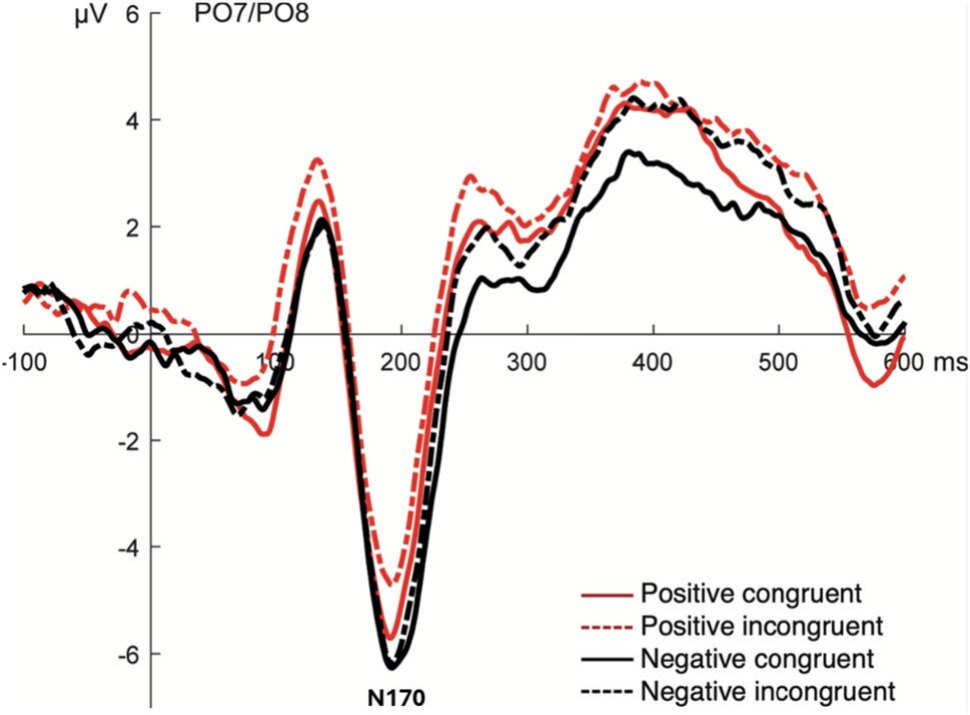
ERP waveform at parietal-occipital electrodes. Grand average of the ERP waveform at parietal-occipital electrodes for the four conditions. The arrow denotes the component

First, we observed an N170 component located at a parietal–occipital region (PO7 and PO8 electrodes) and displaying a negative deflection with a maximum peak at 194 ms (Fig. 3). The statistical analysis for the amplitude revealed a main effect for the emotional valence. In general, negative emoticons elicited larger amplitudes compared with the positive emoticons (*F*(1,19) = 11.68; *p* = 0.003, *η*^*2*^_partial_ = 0.381). An effect was also found for the hemisphere location, whereas the right hemisphere showed an increased amplitude than the left hemisphere (*F*(1,19) = 5.36; *p* = 0.032, *η*^*2*^_partial_ = 0.220). We did not observed significant amplitude differences for congruence (*F*(1,19) = 0.67; *p* = 0.422). Also, we did not observe interaction effects.

#### EPN

**Fig. 4:**
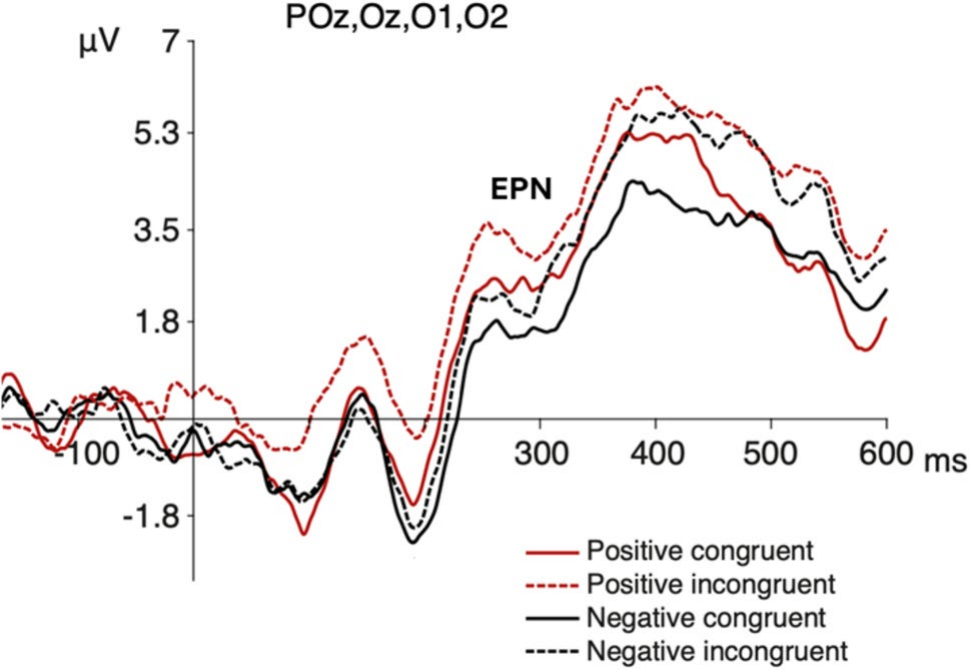
ERP waveform at occipital electrodes. Grand average of the ERP waveform at occipital electrodes for the four conditions. The arrow denotes the component

Further, we observed an occipital negativity (Oz, POz, O1, and O2 electrodes) around 255–285 ms after the emoticon appearance (Fig. 4). We observed a main effect for valence, with a negative waveform larger for negative emoticons (*F*(1,19) = 5.28; *p* = 0.033, *η*^*2*^_partial_ = 0.218). In this case, no significant effects were observed for congruence (*F*(1,19) = 1.33; *p* = 0.264, *η*^*2*^_partial_ = 0.065), and we did not observe interaction effects.

**Fig. 5:**
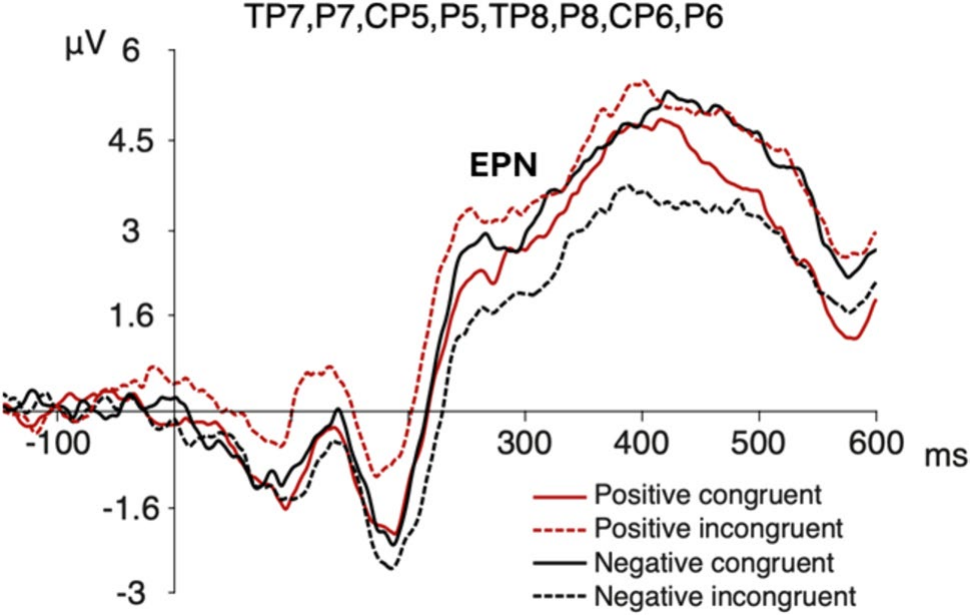
ERP waveform at parieto-temporal electrodes. Grand average of the ERP waveform at parieto-temporal electrodes for the four conditions. The arrow denotes the component

In addition, we detected a parieto-temporal negativity within the same time window (Fig. 5). The statistical analysis of this waveform showed a main effect for congruence (*F*(1,19) = 6.27; *p* = 0.022, *η*^*2*^_partial_ = 0.248), with a larger negativity for the congruent rather than the incongruent conditions. We did not observe significant differences for valence (*F*(1,19) = 1.612; *p* = 0.22) or hemisphere (*F*(1,19) = 1.484; *p* = 0.238) factors. Interaction effects were not detected.

### Mass univariate approach and permutation results over the EPN component at different regions

**Fig. 6:**
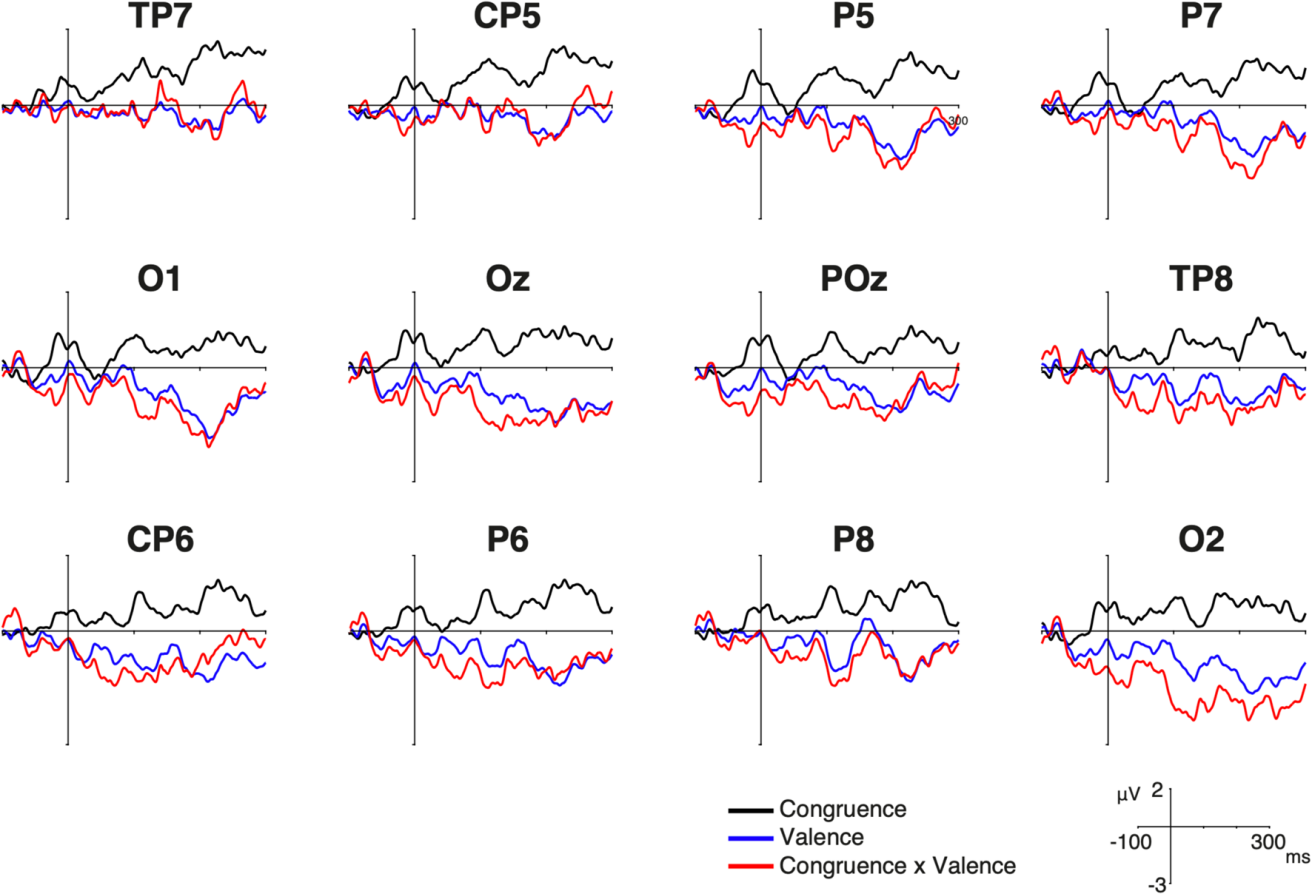
Main effects at parieto-temporal and occipital electrodes. Congruence: Incongruent minus congruent ERP waveform. Valence: Negative minus positive ERP waveform. Congruence × valence: negative minus positive ERP waveform in incongruent condition

**Fig. 7:**
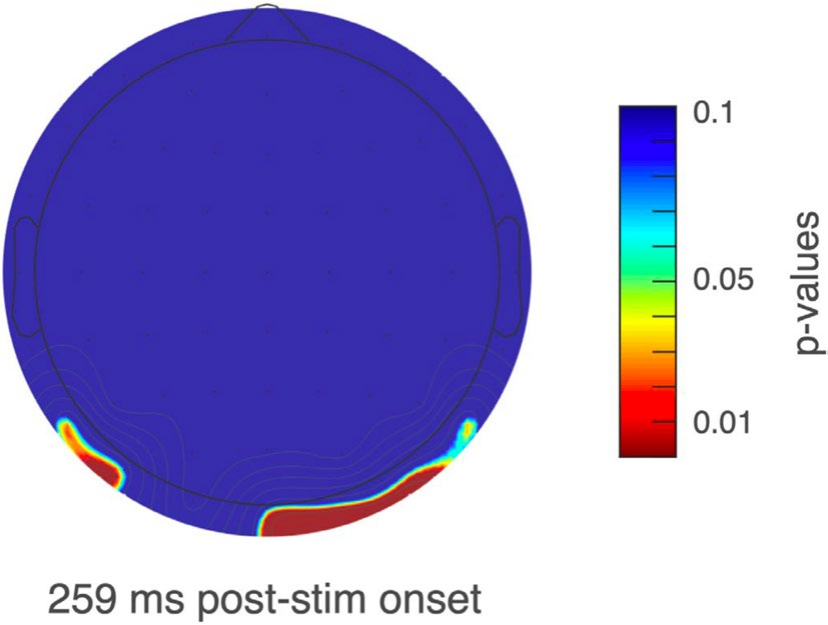
Cluster topography relative to EPN time-window. Topoplot illustrates significant interaction effect between valence and congruence at occipital sites, according to permutation test based on the cluster mass statistic. Heat map represents the distribution of *p* values for the 64 electrodes to specific latency relative to EPN time-windows

We performed a mass univariate analysis to evaluate whether the occipital EPN and parieto-temporal modulations within the same time-window are related. We found a significant main effect for the factor congruence in this time-window. This effect was widely distributed over the scalp, but it did not include the electrodes sites classically observed in EPN analyses (Oz, POz, O1 and O2). At these sites, we detected a significant interaction around 259 ms between valence and congruence (see Figs. 6 and 7; see Supplementary Fig. 1 for the larger congruence x valence interaction effect, and the Supplementary Figs. 2–4 for the significant effect matrices across electrodes). These results suggest that these two modulations are related each other reflecting an interaction between valence and congruence at this early stage of processing.

## Discussion

We investigated the emotional contextual influence in the early brain processing of typographic emoticons using an emotional congruence judgment task. In general, our study provides behavioral and electrophysiological evidence that typographic emoticons are processed similarly than faces, and that explicitly attended congruent contexts enhance the emotional salience during the early stages of processing. Our behavioral results show that the context valence interacts with the valence of the emoticons, suggesting that the contextual emotional information influences their processing (Wieser and Brosch 2012). We observed that positive emoticons had higher accuracy rates and faster RTs than negative emoticons during the congruent condition. More specifically, the RTs during the congruent condition are faster than during the incongruent condition for positive emoticons, when compared to emoticons with a negative valence. On the contrary, the incongruent condition had better accuracy and faster RTs when the emoticons were negative. Our behavioral results also support previous studies reporting higher performance rates for congruent happy faces (Aguado et al. 2013; Diéguez-Risco et al. 2015; Righart and de Gelder 2008b). Altogether, these results suggest an influence of emotional linguistic context on judging congruence performance when they have positive emotions.

Some studies have supported the hypothesis that positive emotions are easier to process than negative ones, when they are congruent with the contextual information. For example, Righart and de Gelder (2008b) presented emotional faces that could be congruent or incongruent with pleasant and unpleasant scenes. They observed a valence effect, with faster RTs for happy faces than for scared faces. Additionally, they observed an interaction between the expression of the face and the context, with faster RTs when the happy faces were presented in happy or neutral contexts, as compared to when they were presented in the frightful contexts. Similarly, we observed faster RTs and better accuracy rates for positive emoticons than for negative ones and, as a whole, for congruent trials than for incongruent ones. In the congruent condition, positive emoticons were responded faster and more accurately than negative ones, but they did not differ in the incongruent condition. This pattern may suggest that emoticons with negative valence are harder to recognize and demand more cognitive effort during their processing. Interestingly, negative emoticons elicited faster RT and better accuracy in the incongruent condition. These results are similar to those obtained by Diéguez-Risco et al. (2015), where the worst performance was observed in congruent negative emotional stimuli, obtaining shorter RTs for incongruent trials. Diéguez-Risco et al. (2015) also suggested that low accuracy rates in negative congruent conditions are related to the notion that recognition of negative faces is easier when they appear within inappropriate situations (Diéguez-Risco et al. 2015). Similarly, other studies have shown that negative emoticons do not have the same effect on the emotional interpretation in incongruent messages, indicating that the emotional meaning depends on the interaction between the valence of the context and the valence of the emoticons (Aldunate et al. 2018a, b; Beyersmann et al. 2022; Walther and D’Addario 2001).

The electrophysiological analysis shows a larger right lateralized N170 for negative emoticons than for positive emoticons, as well as a significant effect for congruence and valence, and the interaction effect of congruence and valence during the EPN time window at different sites. Previous studies observed a right lateralization for the N170 component evoked by emoticons, as those classically reported for human faces (Cao and Zhao 2018; Churches et al. 2014) in agreement with our current results. Furthermore, the lateralization of the N170 depends on the category of the stimulus (e.g. Bentin et al. 1999). Linguistic stimuli evoke greater N170 amplitudes in the left hemisphere, while objects evoke no hemispheric differences (e.g. Rossion et al. 2003). Since the right N170 lateralization has been associated with the whole face processing (Bentin and Deouell 2000; Calvo and Beltrán, 2014; Carbon et al. 2005; Schweinberger et al. 2002), our results support the notion that the typographic emoticons during text-based communication are holistically processed as faces, rather than objects or linguistic punctuation marks. The observed main effect of emotional valence, with a larger N170 component evoked by emoticons with negative valence, indicates that the affective valence of emoticons presented within a context is integrated at these early processing stages, even though emoticons are schematic representations of faces displayed horizontally. This emotional valence effect for emoticons supports that the early perceptual processing mechanisms associated with N170 integrate the information from several sources, such as facial expressions and emotional information (George 2013; Hinojosa et al. 2015; Rossion and Jacques 2011). Accordingly, the activation of the inferotemporal cortex has been related to the simultaneous processing of faces and emotional information (Kawasaki et al. 2012; Xu and Biederman 2010).

Several studies have shown emotional effects over the N170 component evoked by negative emotional faces (Batty and Taylor 2003; Eger et al. 2003). However, these modulatory effects are not entirely consistent among all studies (Hinojosa et al. 2015). Whereas some studies have reported larger N170 amplitudes for faces with negative valences (i.e., fearful and angry faces) (Batty and Taylor 2003; Krombholz et al. 2007; Lynn and Salisbury 2008; Zhao and Li 2006), other studies have found larger amplitudes for positive emotional expression (happy faces) (Marinkovic and Halgren 1998; Rossignol et al. 2012; Schacht and Sommer 2009). In our study, we observed an enhanced N170 amplitude for negative emoticons while people are judging their congruity, suggesting that the presentation of negative emoticons may require more attentional resources; ultimately evoking larger N170 amplitudes, and reflecting an enhanced perceptual processing (Churches et al. 2010; Hillyard et al. 1998; Vuilleumier and Schwartz 2001).

Furthermore, we did not observe contextual influences during the modulation of the early N170 component amplitude. In agreement with Krombholz et al. (2007), we observed the same emotional effect of N170, with larger amplitudes for emoticons with negative expressions, with no congruence effects. Accordingly, Dozolme et al. (2015), using a congruence judgment task, did not find contextual effects on the N170 modulation. However, with a similar paradigm, Diéguez-Risco et al. (2015) reported a congruence effect in the modulation of the N170 component, with larger amplitudes on incongruent trials than on congruent trials. The authors suggested that the modulation corresponded to enhanced perceptual processing of the incongruent trials and the involvement of top-down processing (Diéguez-Risco et al. 2015). These conflicting results might indicate that it is yet unclear how context influences N170 modulation. Possibly, the differences between studies lie on the features of the emotional stimuli, on the proportion of stimuli with negative and positive valence between conditions, and/or on instructions in the tasks and attentional focusing (Wieser and Brosch 2012). Additional studies are needed to allow conclusive interpretations on the contextual influence in N170 modulations.

Our study also found modulatory effects on the time window of 255–285 ms at occipital electrodes, classically reported as EPN component. We found an effect for the valence of emoticons at occipital sites, with larger amplitudes for negative ones. This EPN modulation has been previously reported (Junghöfer et al. 2006; Langeslag and Van Strien 2018; Schupp et al. 2004), reflecting a facilitated processing of motivationally significant visual stimuli in relationship with their emotional salience (Schupp et al. 2006a, b). The larger negative amplitude of the EPN component for negative stimuli, has also been interpreted as an enhanced motivational significance for threatening or negative stimuli (Schupp et al. 2004; Yoon et al. 2016). Apparently, the congruence judgement task does not affect this modulation.

Contextual influences has been observed as modulations of the amplitude of the EPN component using negative social contexts and neutral faces (Klein et al. 2015; Wieser et al. 2014). We did not find such modulation at occipital EPN, however, we observed a congruence effect with an enhanced negativity for congruent emoticons over parieto-temporal sites at the same EPN latency. This early congruence effect is also consistent with a similar modulation observed at the same latency and scalp topography, referred previously as early-N400 (Diéguez-Risco et al. 2015; Dozolme et al. 2015). The classic modulation of the N400 component is observed during semantic incongruency in parietal sites with a latency around 400 ms post-stimulus (Kutas and Federmeier 2011). Conversely, the early-N400 component appears to be larger during congruent conditions and their latency resembles the temporal window of the EPN component. The fact that this component has been reported during emotional congruency judgment tasks, as well as its temporal and topographical proximity with the EPN component, raises important questions regarding its functional significance and nature (Aldunate et al. 2018b). Consequently, we propose that this inverted congruence effect might not be necessarily independent from the process reflected by EPN modulation. In fact, in a follow up mass univariate permutation analyses, we found an interaction between congruence and valence at occipital and parieto-temporal sites. The congruence effect at parieto-temporal sites, and the congruence x valence interaction observed in the mass univariate analysis, suggest a relationship between congruence and valence processing at these early stages. This relationship might reflect that the emoticon’s emotional significance, as observed as modulations in the EPN component, increases according to the congruence between the emoticon’s valence and the valence of the embedded text message, as observed as modulations in the parieto-temporal component. Thus, the congruent contextual information might facilitate the allocation of attentional resources in the processing of the emoticons at the early stages of perceptual processing (Aldunate et al. 2018b). More specifically, the expectations triggered by the text message creates a top-down modulatory influence affecting the emotional significance of the emoticons. Further studies should consider appropriate designs to test the interareal modulatory effect within this time window.

Our results highlight the expressive character of the emoticons (Feldman et al. 2017). Messages with congruent emoticons are rated as more emotionally intense than when they are without these emotional cues (Derks et al. 2008). While some studies have shown that digital cues such as emojis are useful for the text-based communication (Thompson and Filik 2016; Weissman and Tanner 2018), other behavioral studies have found increased processing costs when the emoticon’s meaning carry on conflicting information with the context (Aldunate et al. 2018a, b; Howman and Filik 2020). Accordingly, several electrophysiological studies have shown that this greater cost for the processing of emoticons occurs at later components such as N400, P600 and the Late Positive Potential (LPP) over parietal regions (Gantiva et al. 2019; Pfeifer et al. 2022; Tang et al. 2020; Weissman and Tanner 2018). For example, a recent study has shown that incongruent emojis (pictograms depicting emotional expressions) have a larger modulatory effect than words at later ERPs, such as the N400 component (Tang et al. 2020), suggesting that the higher-order integration processing indexed at 400 ms latencies demands increasing allocation of cognitive resources when they are preceded by incongruent contexts (for a review, see Kutas and Federmeier 2011). Conversely, our results suggest that the context also exerts early modulatory effects during the processing of the emoticons that are congruent with the emotional meaning of the context. Future research should investigate if the observed congruence effect in EPN time-window for emoticons is related to the congruence judgement task or if it is also present in other tasks as emotion recognition (Aguado et al. 2019).

In summary, we aimed to determine how the emotional valence of emoticons and the preceding linguistic context influences the processing of emoticons as a face, analyzing the modulation of the N170 component. On the other hand, how the emotional valence of the preceding linguistic context influences the emotional salience of the emoticon at early stages of perceptual processing by facilitating attentional resources allocation, thus modulating the EPN. Our results suggest that the processing of typographic emoticons in text-based communication impact at these two stages. First, we show that emoticons are processed similarly to faces, therefore evoking an N170 component. Also, their emotional valence modulates the amplitude of this component with a bias towards emoticons with negative valence. Importantly, ERP studies on face processing have focused on faces in upright and inverted positions (rotated 180 degrees), showing that inverted faces tend to generate a higher amplitude and longer latency N170 component. This has been related to increased difficulty processing for faces that are not in their usual orientation (Itier and Taylor 2004; Rossion and Gauthier 2002). However, there is a lack of ERP studies for "lying down" faces (rotated 90 degrees). In the study of Churches et al. (2014), it was observed that lying down emoticons evoked a greater amplitude and latency of the N170 component compared to real faces in their upright position, suggesting that, although they are processed as faces, emoticons are more difficult to process.

Second, EPN modulations show the classically reported effect for emotional valence over occipital sites (larger negativities for negative emoticons) together with a parieto-temporal negative deflection, which is larger for congruent emoticons. These findings suggest that the contextual emotional information facilitates the allocation of attentional resources and increases the emotional significance of congruent emoticons, probably influencing their perceived expressivity. These findings suggest that EPN modulations might be generated by different processes that involve spatially segregated topographies in the posterior regions of the scalp. To our knowledge, this spatial segregation of such processes has not been reported before. Further studies are needed to determine the nature and potential relation between the emotional salience and contextual congruence and their electrophysiological correlates.

Importantly, although in our design we consider previous studies that show that it is more common for text messages to precede the presentation of emoticons (e.g., Provine et al. 2007), our study has some limitations that must be taken into account. Specifically, we recognize that in daily life emoticons and sentences are often presented simultaneously, so our experimental design, which presents these elements sequentially and in isolation, may not fully reflect that reality. While we understand that this difference in presentation may affect the generalizability of our findings, our study was designed to decompose and analyze the specific components of early emoticons processing in relation to their emotional congruency. Although this approach allowed us to obtain more detailed and controlled data, we are aware of the limitations it implies in terms of ecological validity. Future studies of N170 and EPN amplitude modulations in early emoticons processing should consider the possibility of implementing experimental designs where emoticons and sentences are presented simultaneously to better evaluate the applicability of the results to everyday experience. Additionally, the cultural sensitivity of emoticons processing should also be addressed in future studies. A multicultural collaborative study would be usefull to especify the general aspects of emoticons processing, and those only present in specific cultures and languages.

## Supplementary Information

Below is the link to the electronic supplementary material.

Supplementary file1 (PDF 701 KB)
