# Supplementary material for: Emotional text messages affect the early processing of emoticons depending on their emotional congruence: evidence from the N170 and EPN event related potentials
Source: Cogn Process. 2024 Aug 24;25(4):621–34. doi: 10.1007/s10339-024-01223-y (PMC11541363; doi:10.1007/s10339-024-01223-y)
Supplement: Supplementary file 1 — Supplementary file1 (PDF 701 KB) [file 10339_2024_1223_MOESM1_ESM.pdf]

SENTENCES: EMOTIONAL SITUATIONS

**Positive Situations**	**Negative Situations**
Tengo una pareja hermosa	Mi papá se cayó por las escaleras
Mi mamá se ganó la lotería	No tengo ningún amigo de verdad
Me saqué un 7,0 en la prueba de álgebra	Esa familia no tiene con qué comer
Mi familia está más unida que nunca	Este mes no me pagaron
Me voy al Caribe	Tengo mucho trabajo atrasado
Mi guagua es hermosa	Mi novio me va a dejar
Mi salud es excelente	Se perdió mi perro
Conocí a alguien muy especial	No encuentro mi billetera
Pasé un excelente fin de semana junto a mi familia	Me echaron del trabajo
Dijeron que mi idea es la mejor	Hoy es mi cumpleaños y nadie se acordó
Nunca había tenido tanta suerte	Se me rompió el computador
Me fue súper bien en el examen	Cada vez tengo más estrés
Entregué mi tesis	No tengo dinero para comer hoy
Quedé primero en la carrera	Estoy pasando un momento muy difícil
La fiesta estuvo buenísima	Me traicionaron
Esta mañana, nació mi hijo	Mi jefe me humilló en público
Me regalaron un auto nuevo	Se murió un familiar cercano
Me gané unas entradas para ver a mi grupo favorito	En mi casa están todos en contra mío
Me dijo que me quería	Reprobé casi todos los exámenes finales
Me ascendieron en el trabajo	Me pusieron una multa
Me subieron el sueldo	Me saqué un 1,0 en la prueba de historia
Son mis mejores amigos	Me fracturé un tobillo
Dormí muy rico anoche	Mi pareja me dejó
Me encantó el regalo que me hiciste	Me chocaron el auto
Voy a comer mi comida favorita	Me duele mucho la cabeza
Ya tengo los pasajes para mi viaje	No creo que logre la nota necesaria para pasar
Salí de vacaciones	Se borró mi tesis
Tengo los mejores amigos del mundo	Perdí el avión
Tomé la mejor decisión de mi vida	Me dijeron que tengo una enfermedad grave
Me felicitaron en el trabajo	Me robaron la bicicleta

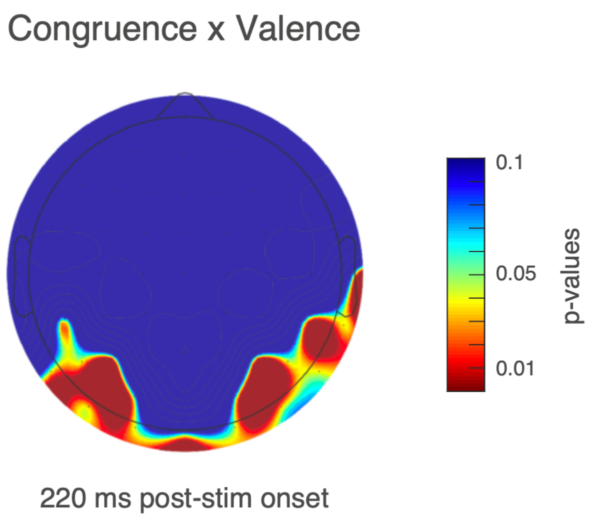

Supplementary Figure 1: Cluster topography of the larger congruence x valence interaction effect latency. Topoplot shows the significant main effect for the congruence x valence interaction according to a permutation test based on the cluster mass statistic. Heat map represents the distribution of p-values for the 64 electrodes.

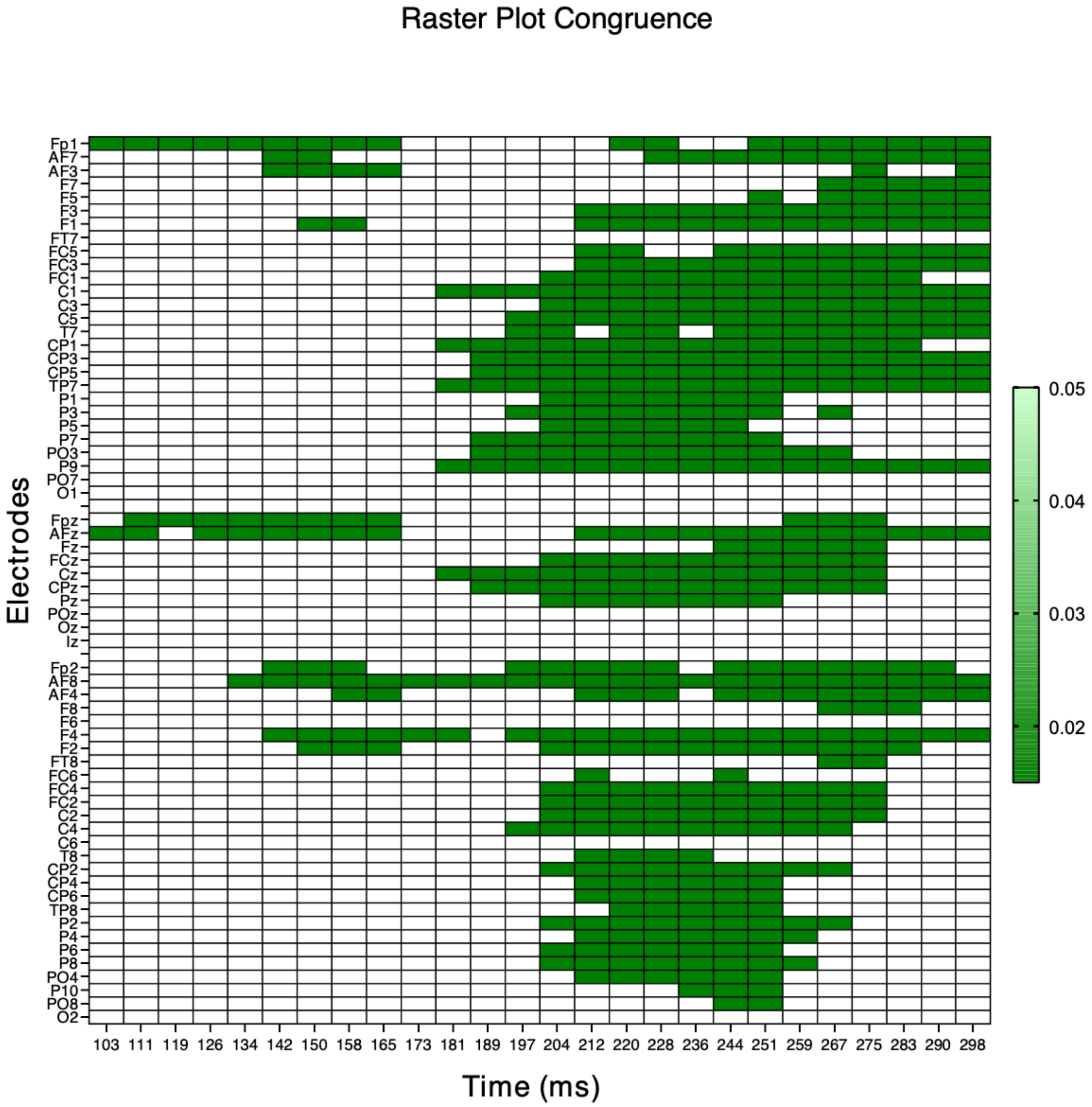

Supplementary Figure 2: Raster diagram illustrating significant main effect for the factor congruence according to permutation test based on the cluster mass statistic. Each colored electrode/timepoint represents a significant p value (p <.05). Gray rectangles indicate electrodes/time points at which no significant effect was found. Note that the electrodes are organized along the y-axis somewhat topographically. Electrodes on the left and right sides of the head are grouped on the figure's top and bottom, respectively.

Midline electrodes are shown in the middle. Within those three groupings, y-axis top-to-bottom corresponds to scalp anterior-to-posterior.

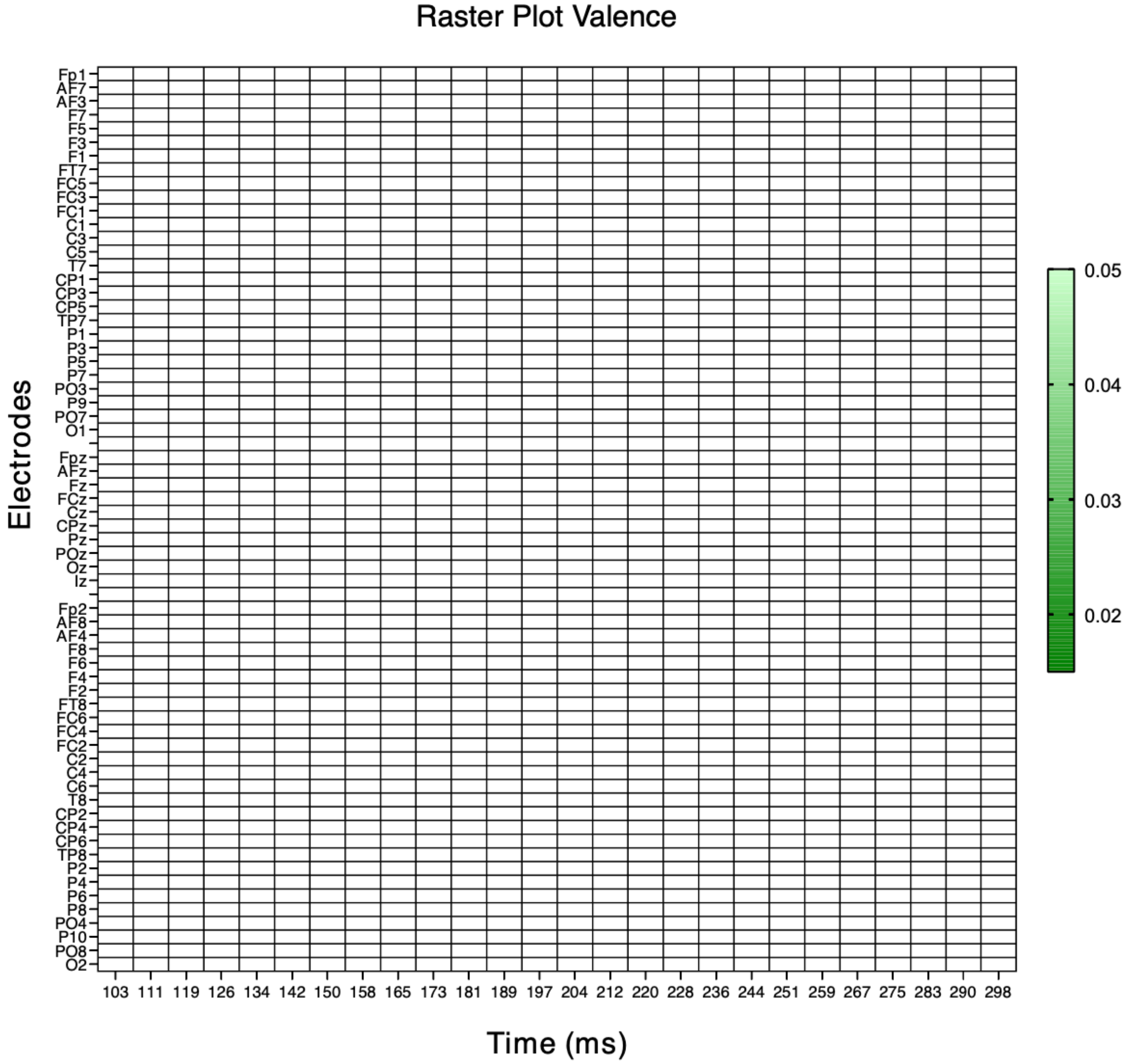

Supplementary Figure 3: Raster diagram illustrating significant main effect for the factor valence according to permutation test based on the cluster mass statistic. Gray rectangles indicate electrodes/time points at which no significant effect was found. Note that the electrodes are organized along the y-axis somewhat topographically. Electrodes on the left and right sides of the head are grouped on the figure's top and bottom, respectively.

Midline electrodes are shown in the middle. Within those three groupings, y-axis top-to-bottom corresponds to scalp anterior-to-posterior.

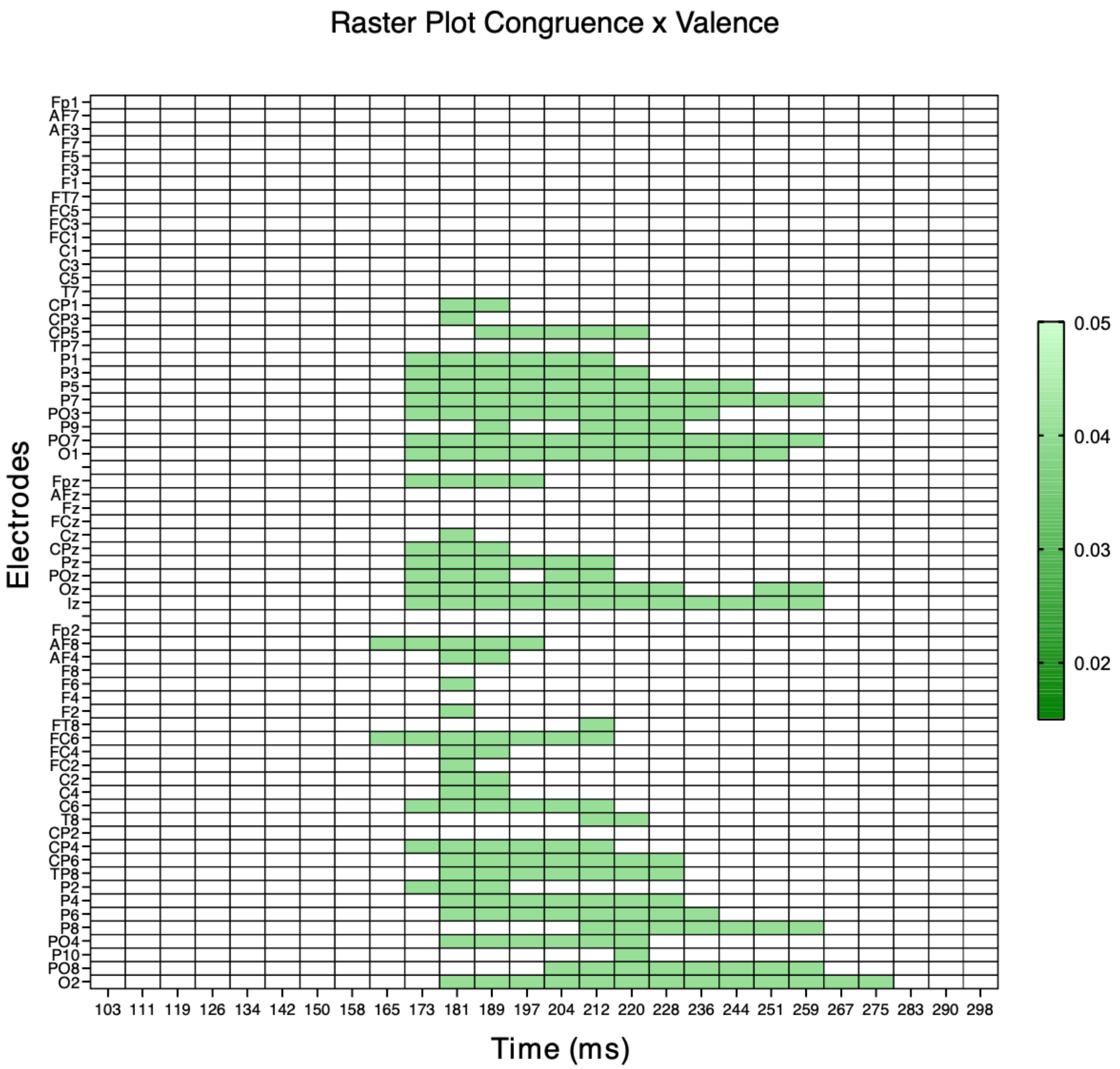

Supplementary Figure 4: Raster diagram illustrating significant interaction between the congruence and valence factors according to permutation test based on the cluster mass statistic. Each colored lectrode/timepoint represents a significant p value (p <.05). White rectangles indicate electrodes/time points at which no significant effect was found. Note that the electrodes are organized along the y-axis somewhat topographically. Electrodes on the left and right sides of the head are grouped on the figure's top and bottom, respectively.

Midline electrodes are shown in the middle. Within those three groupings, y-axis top-to-bottom corresponds to scalp anterior-to-posterior.
